# NO_3_^−^/H^+^ Antiport in the Tonoplast of Cucumber Root Cells Is Stimulated by Nitrate Supply: Evidence for a Reversible Nitrate-Induced Phosphorylation of Vacuolar NO_3_^−^/H^+^ Antiport

**DOI:** 10.1371/journal.pone.0073972

**Published:** 2013-09-11

**Authors:** Magdalena Migocka, Anna Warzybok, Anna Papierniak, Grażyna Kłobus

**Affiliations:** Wrocław University, Institute of Experimental Biology, Department of Plant Molecular Physiology, Wrocław, Poland; Iowa State University, United States of America

## Abstract

Studies in the last few years have shed light on the process of nitrate accumulation within plant cells, achieving molecular identification and partial characterization of the genes and proteins involved in this process. However, contrary to the plasma membrane-localized nitrate transport activities, the kinetics of active nitrate influx into the vacuole and its adaptation to external nitrate availability remain poorly understood. In this work, we have investigated the activity and regulation of the tonoplast-localized H^+^/NO_3_^−^ antiport in cucumber roots in response to N starvation and NO_3_^−^ induction. The time course of nitrate availability strongly influenced H^+^/NO_3_^−^ antiport activity at the tonoplast of root cells. However, under N starvation active nitrate accumulation within the vacuole still occurred. Hence, either a constitutive H^+^-coupled transport system specific for nitrate operates at the tonoplast, or nitrate uses another transport protein of broader specificity to different anions to enter the vacuole via a proton-dependent process. H^+^/NO_3_^−^ antiport in cucumber was significantly stimulated in NO_3_^−^-induced plants that were supplied with nitrate for 24 hours following 6-day-long N starvation. The cytosolic fraction isolated from the roots of NO_3_^−^-induced plants significantly stimulated H^+^/NO_3_^−^ antiport in tonoplast membranes isolated from cucumbers growing on nitrate. The stimulatory effect of the cytosolic fraction was completely abolished by EGTA and the protein kinase inhibitor staurosporine and slightly enhanced by the phosphatase inhibitors okadaic acid and cantharidin. Hence, we conclude that stimulation of H^+^/NO_3_^−^ antiport at the tonoplast of cucumber roots in response to nitrate provision may occur through the phosphorylation of a membrane antiporter involving Ca-dependent, staurosporine-sensitive protein kinase.

## Introduction

Nitrate variation in soils strongly influences plant growth and development, so plants had to evolve mechanisms allowing them to react and respond to frequent changes of the NO_3_^−^ level. Namely, plants acquire NO_3_^−^ ions through different types of uptake systems operating in their roots: low (LATS) and high (HATS) affinity transport systems which have both constitutive and inducible components [1]-[6]. All four nitrate transport systems coexist within the cell and each exhibits different sensitivity to nitrate and operates at a different external NO_3_^−^ concentration range. The constitutive high-affinity transport system (cHATS) operates at low external nitrate concentrations and is complemented by the inducible high-affinity system (iHATS), which is activated by very low NO_3_^−^ supply [3], [7]. When nitrate supply reaches greater values, the low affinity system (LATS) takes over [8], [9]. For the last decades much attention has been given to understanding how these transport systems are regulated at the molecular level. The passage of NO_3_^−^ through the plasma membrane of root cells has been particularly intensively studied. It is well known that nitrate uptake into the root cells occurs via energy dependent symport with two protons translocated along with one NO_3_^−^ ion against transmembrane gradient of nitrate [10], [11]. The energy for this process comes from the electrochemical gradient generated by plasma membrane proton pump H^+^-ATPase [10]–[12]. In the plasma membranes of root cells a few transporters belonging to NRT1 (Nitrate transporter 1) and NRT2 (Nitrate transporter 2) families use the energy provided by H^+^-ATPase to transport nitrate into the cells. Two *Arabidopsis* NRT1 proteins (AtNRT1.1 and AtNRT1.2) and two NRT2 proteins (AtNRT2.1 and AtNRT2.2) are directly involved in nitrate uptake but they participate in different-affinity uptake systems [13]–[16]. AtNRT2.1 and AtNRT2.2 are involved in high-affinity NO_3_^−^ influx, whereas AtNRT1.2 is a low-affinity nitrate transporter [4], [14], [17]. AtNRT1.1 functions as a dual-affinity transporter: the phosphorylated form of the protein is involved in high-affinity nitrate uptake, whereas the dephosphorylated form contributes to low-affinity NO_3_^−^ influx [5], [6], [18], [19]. The posttranslational modification relying on phosphorylation/dephosphorylation of the nitrate transport proteins is not surprising since the putative amino acid sequences of NRT1 and NRT2 families contain a number of conserved protein kinase C recognition motifs in their N- and C-terminal domains or in the central loops [20]. This may indicate the importance of phosphorylation and dephosphorylation events in the regulation of other members of NRT families.

Although the mechanism of regulation of nitrate influx into the cell has been extensively studied, the way by which nitrate sequestration within the vacuole is controlled is still unknown, and the question arises whether similar regulation mechanisms occur for the tonoplast and plasma membrane nitrate transport activities. It was earlier shown that plants provided with unlimited supplies of NO_3_^−^ stored most root and shoot nitrate within the vacuoles [21]. Nitrate was also accumulated in the central vacuole of leaf cells [22] or root tip cells [23] and the vacuolar NO_3_^−^ concentration increased in response to high external nitrate concentrations [2], [23]. The mechanism of NO_3_^−^ transport across the tonoplast has been recently more extensively studied. It was earlier assumed that since the Δψ between the cytosol and the vacuole is not sufficient to drive the NO_3_^−^ accumulation within the vacuole, the flux of nitrate across the tonoplast must occur via both passive and active ways. Indeed, the experiments using direct and indirect assay of NO_3_^−^ or H^+^ transport have provided evidence for an NO_3_^−^/H^+^ antiporters in the tonoplast [24]–[27]. There are two proton pumps (H^+^-ATPase and H^+^-PPase) on tonoplast which pump H^+^ into vacuole and thus contribute to building an electrochemical proton gradient between cytoplasm and vacuolar lumen [28]–[30]. The pH gradient generated by both pumps serves as a driving force for ion antiport at tonoplast membrane [30]. V-ATPases are highly conserved, multisubunit proton pumps that are built of two domains: the membrane-integral V0 complex of subunits VHA-a, -c, -c′, -c′′, -d, and –e, which is responsible for proton translocation into the lumen of endomembrane compartments and the peripheral V1 complex of subunits VHA-A to -H, involved in ATP hydrolysis [31]. In contrast, the VPPase functions as a homodimer of a single polypeptide, and uses energy from the pyrophosphate (PPi) hydrolysis to drive proton transport across membranes [32], [33]. However, since PPi is a by-product of several biosynthetic processes, it has been suggested that the V-PPase may be the major proton pump responsible for the acidification of vacuoles of young, growing cells [34]. It has also been proposed that V-PPase may function as a supporting system for V-ATPase under stressful conditions limiting the availability of ATP [33]. Despite the functional relation of these two pumps remains to be established, it has been already shown that a proton motive force generated by V-ATPase can be used to drive active transport of nitrate ions into tonoplast membranes isolated from cucumber roots [35]. Moreover, it was also demonstrated that nitrate accumulation within vacuoles of the *Arabidopsis* mutants lacking V-ATPase at tonoplast was significantly reduced despite the presence of V-PPase at vacuolar membrane [36]. Although V-PPase was sufficient for survival of the mutant under certain conditions, tonoplast V-ATPase activity was required for efficient nitrate storage and metal sequestration, suggesting the ATP-dependent pump may be the main contributor to active nitrate antiport into vacuoles [36].

The research on the molecular identity of antiporters that use the electrochemical gradient generated by V-ATPase to drive nitrate transport into vacuole are now in progress. The evidence has been presented that some proteins of the chloride channel family (CLC) reside at tonoplast and act as NO_3_^−^/H^+^ exchangers [18], [37], [38]. Specific nitrate transporters (NRT1 and NRT2) may also be implicated in nitrate accumulation into the vacuole. It was shown that tonoplast-localized AtNRT2.7 protein may be important for nitrate loading into the vacuole of dry seeds during seed maturation [39]. Since nitrate transporters are encoded by multigene families and the information about the localization of nitrate transporters in other plants is still scarce, we cannot exclude the contribution of other proteins of both NRT families in nitrate accumulation within the vacuole.

Although protein candidates have been proposed that could catalyze active nitrate influx into the vacuole, the regulation of this process remains poorly understood. The storage of nitrate within vacuoles is significant for both osmotic purposes and redistribution to the cytoplasm under starvation [22]. Since active accumulation of NO_3_^−^ in the vacuole under unlimited NO_3_^−^ supply may be considered as one of the mechanisms enabling plants to survive further NO_3_-limited conditions, it is highly desirable to understand the regulation of this process. In this work, we try to elucidate the mechanism of regulation of nitrate accumulation in tonoplast membranes isolated from cucumber root cells. Our studies on the effect of nitrate, nitrogen induction or nitrogen starvation on the rate of NO_3_^−^ transport across the tonoplast reveal that nitrate itself may regulate the activity of nitrate transporters localized at the vacuolar membrane. We also show that phosphorylation/dephosphorylation events may be involved in regulation of the nitrate transport process leading to NO_3_^−^ accumulation within the vacuole lumen.

## Materials and Methods

### Plant Material

All experiments were performed using the roots of 7-day-old seedlings of *Cucumis sativus* L var. Krak. Seeds were germinated in darkness and then grown on nutrient solution, pH 6.0, with or without nitrate. The experimental material included plants grown on NO_3_^−^-rich (NO_3_^−^-grown plants) or N-free (N-deprived plants) medium for 7 days and plants grown on N-free medium for 6 days and transferred on NO_3_^−^-rich medium for 24 hours (NO_3_^−^-induced plants). The standard medium contained the following macroelements: 1 mM K_2_SO_4_, 0.7 mM CaSO_4_, 0.33 mM Ca(H_2_PO_4_)_2_, 0.33 mM MgSO_4_ with 5 mM KNO_3_ (NO_3_^−^-rich medium) or 2.5 mM K_2_CO_3_ (N-free medium). Microelements added to the medium included: 25 µM ferric citrate, 3 µM MnSO_4_, 1.7 µM H_3_BO_3_, 0.3 µM CuSO_4_, 0.003 µM ZnSO_4_ and 0.017 µM Na_2_MoO_4_. All plants were grown under a 16-h photoperiod (180 mmol m^−2^ s^−1^) at 25°C during the day and 22°C during the night.

### Tonoplast Isolation

The membranes and the soluble (cytosol) fraction were isolated after homogenization of fresh roots in 70 mM TRIS-MES (pH 8.0), containing 500 mM sorbitol, 10 mM sodium glycerophosphate, 280 mM choline chloride, 2 mM salicylhydroxamic acid, 3 mM Na_2_EDTA, 26 mM K_2_S_2_O_5,_ 5 mM DTT, 0.2% BSA and 0.5% PVPP, followed by 15-min centrifugation at 10 000 g. The total microsomal membranes were collected through the subsequent ultracentrifugation at 80 000 g for 30 min. The supernatant obtained after this centrifugation was used as a soluble cytosolic fraction. Vacuolar membranes were separated by centrifugation of microsomes on a discontinuous sucrose density gradient (20, 28, 32 and 42% w/w) as described earlier [35]. The fraction enriched in tonoplast displaying the highest activity of PPase and V-ATPase (band of 20% sucrose) was diluted 4 times with 5 mM Tris-MES (pH 7.2), 1.1 M glycerol, 1 mM Na_2_EDTA and 1 mM DTT, and further recentrifuged at 80 000 g for 30 min. The final pellet of vacuolar membranes was resuspended in a 20 mM Tris-MES containing 250 mM sucrose and immediately used to determine transport activity. The high purity and the right-side out orientation of tonoplast membranes were determined earlier [40].

### Assays of ATP Hydrolysis and ATP-dependent Proton Transport

The rate of ATP hydrolysis was determined from the differences in the rate of Pi release in the absence and presence of specific V-ATPase inhibitor 50 mM KNO_3_. Inorganic Pi release was determined spectrophotometrically as described by Gallagher and Leonard [41] and Ames [42]. ATP-dependent proton transport activity was measured as a drop of acridine orange absorbance at 495 nm as described earlier [35]. After 5 min incubation of 50 µg vesicle protein with 20 mM TRIS-MES (pH 7.2), 0.25 M sucrose, 50 mM KCl, 1 mM DTT and 10 µM acridine orange, the reaction was initiated by addition of 3 mM MgATP. In control assay, 3 mM MgSO_4_ instead of 3 mM MgATP was added to the reaction medium to determine the passive permeability of tonoplast membranes to protons (Figure S1A)_._ In the assay including MgATP, bafilomycin, KNO_3_ and gramicidin were used to confirm that the observed A_495_ changes result from V-ATPase activity (Figure S1B). 5 mM KNO_3_ was added to MgATP-energized vesicles to initiate H^+^-coupled NO_3_^−^ influx into membranes accompanied by the increase in acridine orange absorbance at 495 nm, in order to confirm the presence of nitrate antiport in tonoplast isolated from cucumber root cells (Figure S2).

### Assays of ΔpH-dependent Proton Transport

To determine anions antiport activity in vacuolar membranes tonoplast vesicles were suspended in pH 6.0 (to impose transmembrane pH gradient, Figure S3) or 8.0 (to maintain equal transmembrane pH, Figure S3) and introduced into 20 mM TRIS-MES (pH 8.0), containing 0.25 M sucrose, 1 mM DTT and 10 µM acridine orange (absorbance assay) or 10 µM quinacrine (fluorescence assay, Figure S5). After 3 min incubation, the reactions were initiated by the addition of different concentrations of KNO_3_, K_2_SO_4_ or KCl to the assay and monitored for the next three minutes. The changes in acridine orange absorbance were measured at 495 nm whereas the quinacrine fluorescence was determined with excitation and emission wavelengths of 420 nm and 500 nm, respectively, using a TD-700 fluorometer (Turner Designs).

The results obtained using two different pH-sensitive probes were comparable, hence only the acridine orange absorbance assay was used in the further studies on nitrate antiport activity. To determine whether tonoplast nitrate transporters and proton pump undergo phosporylation/dephosphorylation event, 50 µl of soluble cytosolic fractions, 5 µM staurosporine and 2 µM phosphatases inhibitors (okadaic acid (OA) or cantharidin) or 5 mM EGTA were applied to the reaction media at the beginning of 3-min incubation of vesicles with acridine orange - containing mixture. In the experiments where the inhibitors of protein kinases or phosphatases were used, an equal amount of their solvent, DMSO was applied to control assays. In the experiments including cytosolic fraction, an equal volume of water was added to control assays.

### Analysis of Tonoplast Nitrate content by HPLC

Nitrate accumulation within tonoplast vesicles imposing artificial pH gradient was also determined using HPLC-ion chromatographic assays. The reaction mixture was composed as described above except there were no pH-sensitive probes included. Different concentrations of nitrate were introduced into the assays and after 5 min incubation, the reactions were filtered through 0.45 µm nitrocelulose membranes (Millipore) under vaccum to separate vesicles from the medium. Nitrate content in the filtrate was determined using High Performance Liquid Chromatography (HPLC) according to Thayer and Huffaker [43] using a PARTISIL 10 SAX column, with 30 mM NaH_2_PO_4_ buffer, pH 3.0, as the mobile phase. H^+^-coupled nitrate accumulation within tonoplast vesicles was calculated from the difference between nitrate determined in vesicles imposing pH gradient and nitrate measured in vesicles without transmembrane ΔpH.

### Western Blot

**Figure 1:**
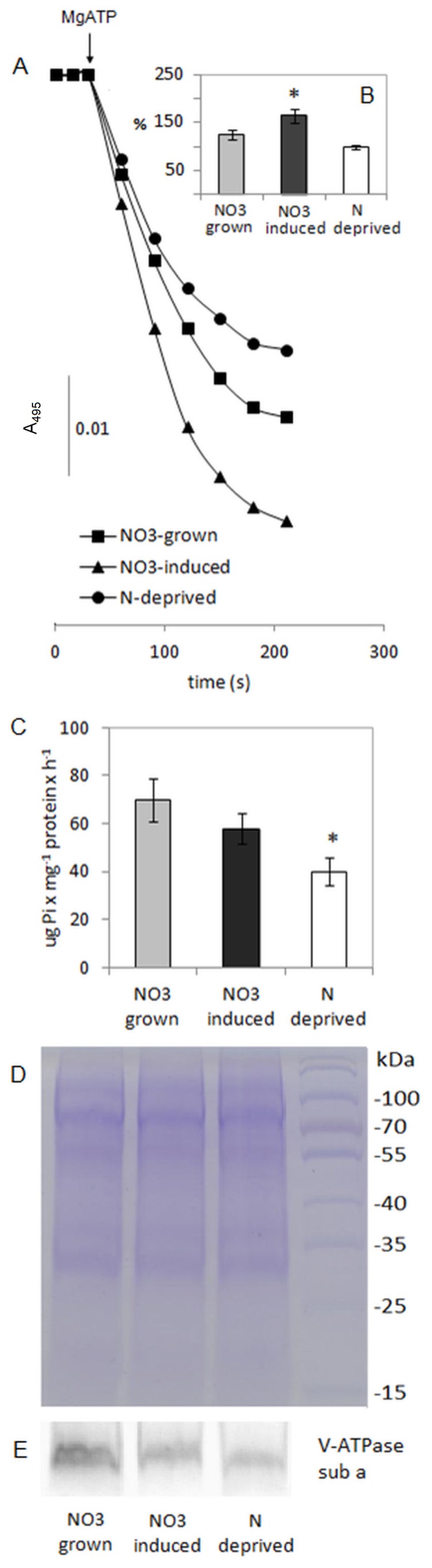
V-ATPase activity and quantity in tonoplast vesicles isolated from roots of cucumber plants grown under different nitrate supply. A–B. The rate of MgATP-dependent, bafilomycin-sensitive proton transport in tonoplast under constant (squares, grey bars) or temporary (triangles, dark bars) NO_3_^−^ supply and under N-deprivation (circles, white bars). Figure A is representative for the results obtained in six independent experiments. Figure B presents the average values ± SD of four independent experiments. Asterisks indicate a significant difference (*P*<0.05) between transport activity of V-ATPase under different nitrate supply. C. NO_3_^−^-sensitive hydrolytic activity in membranes obtained from NO_3_^−^-grown (light grey bars), NO_3_^−^-induced (dark grey bars) and N-deprived (white bars) plants. Data are the average values ± SD of three independent experiments. Asterisks indicate a significant difference (*P*<0.05) between V-ATPase activity under different nitrate supply. D. A representative Coomassie blue-stained gel of total tonoplast protein isolated from plants grown under different nitrate regime. 15 µg of tonoplast proteins were separated by SDS-PAGE on a 10% linear acrylamide gel. The positions of PAGE molecular mass markers are shown in kilodaltons on the right of the gel image. E. Immunoblot of V-ATPase subunit a on tonoplast membranes obtained from plants grown under different nitrate regime. Presented picture is representative for the results obtained in three to four independent experiments.

Membrane protein (15 µg) was mixed with the 10 mM TRIS buffer containing 2% (w/v) SDS, 80 mM DTT, 40% (w/v) glycerol, 5 mM PMSF, 1 mM EDTA, and 0.05% (w/v) bromophenol blue and incubated at room temperature for 30 min. The samples were separated by SDS/PAGE using 10% acrylamide gel (Sigma) and stained with Coomassie Blue (Figure 1D) or transferred onto a nitrocellulose membrane (Schleicher & Schuell BioScience). The membrane was incubated with polyclonal antibodies against the subunit a of tonoplast V-ATPase (Agrisera) at room temperature for 1 h. After repeated washing, the membranes labeled with primary antibodies were further incubated for 1 h with secondary antibodies (conjugated to horseradish peroxidase, Agrisera) and visualized by staining with DAB.

### Protein Determination

Protein was measured using the Bradford [44] method with BSA as a standard.

### Database Searching

The complete genomic sequence from cucumber has recently been made available to the public in GenBank with accession code ACHR01000000 [45]. NCBI database (whole-genome shotgun reads) was used for Blastn searches of cucumber sequences with homology to the previously annotated *Arabidopsis thaliana CLC-a, CLC-c* and *CLC-g* sequences. Full cDNAs as well as the protein sequences encoded by the newly identified cucumber *CLCs* were generated using FGENESH [46] and Eukaryotic GeneMark.hmm [47]. The primers used for PCR and qPCR were designed to amplify highly specific products. The specificity of all PCR products was confirmed through sequencing and the partial ESTs were submitted into GeneBank; they are available under the following accession numbers: JK525488 (*CsCLCa*), JK525489 (*CsCLCc*) and JK525490 (*CsCLCg*).

### Expression Analyses

Total RNA was extracted from cucumber roots with TRI Reagent (Sigma) according to manufacturer’s instructions. The quantity and quality of RNA were determined using a NanoDrop spectrophotometer (Nyxor, ND-100). RNA integrity was checked on 2100 Bioanalyzer (Agilent) using RNA kits and the RNA Integrity Number (RIN) algorithm (Figure S4). Two micrograms of DNAse-digested total RNA were used for cDNA synthesis using random primers and High Capacity cDNA syntesis Kit (Applied Biosystems). 1 µl of cDNA was used as template for standard PCR with Marathon Polymerase (A&A Biotechnology) according to the following programme: 94°C for 5 min followed by 30 cycles of 94°C for 30 s; 56°C for 30 s and 68°C for 1 min, with final extension at 72°C for 5 min. PCR products were ligated to pGEM T-Easy (Promega) and sequenced 3–4 times. The same cDNA used for gene isolation was used for real-time PCR analyses. PCR amplification was carried out using LighCycler 2.0 (Roche) and SYBR Green DNA dye, in a total volume of 10 µl, containing 1 µl of 5-fold-diluted cDNA template, 1 µl of each primer (10 µM), 2 µl of nuclease-free water and 5 µl of 2×Master SYBR® Green I B (A&A Biotechnology). The thermal cycling conditions were 95°C for 10 min followed by 45 cycles of 95°C for 10 s, 56°C for 10 s, 72°C for 15 s. The gene encoding clathrin adaptor complex subunit (CACS) was used as internal control with the following forward 5′-GTGCTTTCTTTCTGGAATGC-3′ and reverse 5′-TGAACCTCGTCAAATTTACACA-3′ primers. A negative control without cDNA template was included in the same PCR run for each primer pair. To confirm the specificity of amplification, melting curve analysis was performed allowing to identify putative unspecific PCR products (e.g., primer dimers, reaction mix contamination) and the RT-PCR products were sequenced. Successive dilutions of the sample with the lowest Cp were used as a standard curve. Amplification efficiency was around 2. For each of the two independent RNA extractions, measurements of gene expression were obtained in duplicate. Specific primers were carefully designed using LighCycler Probe Design Software 2 (Roche): *CLCa* (forward, 5′-TATTAGGGTTATTCACCTTCGGTATC-3′; reverse, 5′-TTCGAGGAATATGACACAAAGAGAAA-3′), *CLCc* (forward, 5′-CTGTAGAGATTTCATTTGATTAATGGTT-3′; reverse, 5′-CATGGAAATGGATGGAGAAATTAAG-3′), and *CLCg* (forward, 5′-TTCTTCTAGTGCAGTAGGATAGAC-3′; reverse, 5′-TTTGAACTGAATTTGCCATTTAACC-3′).

### Statistical Analysis

Data on proton and nitrate transport activities were analysed by one-way completely randomized ANOVA and means comparison was performed by t test at significance level of 0.05. A GraphPad Prism program (GraphPad Software, Inc) was used to fit the data directly to the Michaelis-Menten equation using nonlinear regression and to display data with Lineweaver-Burk plots. The qPCR data were analyzed by the ΔΔCT - method using the LightCycler® Software 4.1 (Roche). Paired student’s *t* test and ANOVA (Excel) were used to confirm statistical significance of difference in *CsCLCs* expression between plants grown under different nitrate supply. The quantitative calculation of Western Blot results was performed using ImageJ software (rsb.info.nih.gov/ij).

## Results

### The Effect of different Nitrate Supply on the Hydrolytic and Transport Activity of Tonoplast V-ATPase

In most research on membrane antiport activities, primary proton pumps have been used to provide the energy to drive ion transport across the lipid bilayer. In the preliminary assay, we studied the effect of varying nitrate availability or deprivation on tonoplast V-ATPase to gain an insight into the rate of proton gradient formation in membranes isolated from plants growing under different nitrate regimes. The generation and maintenance of proton gradient in isolated tonoplast vesicles were monitored using acridine orange as a ΔpH-sensitive probe. Upon addition of MgATP, the formation of a ΔpH in the tonoplast was observed as a decrease of acridine orange absorbance at 495 nm (Figures 1A and 1B, Figures S1A–B). ΔpH generation across membranes was inhibited by bafilomycin (500 nmol) or KNO_3_ (50 mM), specific inhibitors of tonoplast V-ATPase, confirming that the effect resulted from vacuolar pump activity (Figure S1B). The rate of proton gradient formation was significantly different between tonoplast membranes isolated from plants grown under various nitrate nutrition. As shown in Figures 1A and 1B, the highest level of proton gradient was generated in membranes isolated from NO_3_^−^-induced plants. In comparison, the rate of ΔpH formation in tonoplast obtained from NO_3_^−^-grown and N-deprived plants decreased to 75% and 60%, respectively. Despite significant stimulation of proton transport activity of tonoplast V-ATPase, the hydrolytic activity of this enzyme was only slightly affected (decreased to 90%) and the level of proton pump protein was decreased up to 60% in NO_3_^−^-induced plants (Figures 1C and 1E). In comparison, the hydrolytic activity and proton pump protein level in membranes isolated from N-deprived plants were much more reduced to almost 50% and 40%, respectively (Figures 1C and 1E). Therefore, the availability of nitrate in nutrient solution had a significant impact on the amount and activity of tonoplast V-ATPase and consequently on the generation of a transmembrane ΔpH, an essential force for the transport of various compounds, e.g. nitrate, between cytosol and vacuoles. In consequence, the N-deprived plants displayed markedly reduced growth of roots, hypocotyls and cotyledons in comparison to NO_3_^−^-grown seedlings, which even developed the first leaf after 6 day-long cultivation on NO_3_^−^-enriched medium. The rate of antiport activity strictly depends on the magnitude of the proton motive force; hence to equip tonoplast prepared from different plants with the same rate of transmembrane ΔpH, we imposed an artificial pH gradient on the vesicles (Figure S3). Thus, we could measure the direct effect of nitrate availability on vacuolar antiporters.

### Uptake of NO_3_^−^ into Tonoplast Vesicles

**Figure 2:**
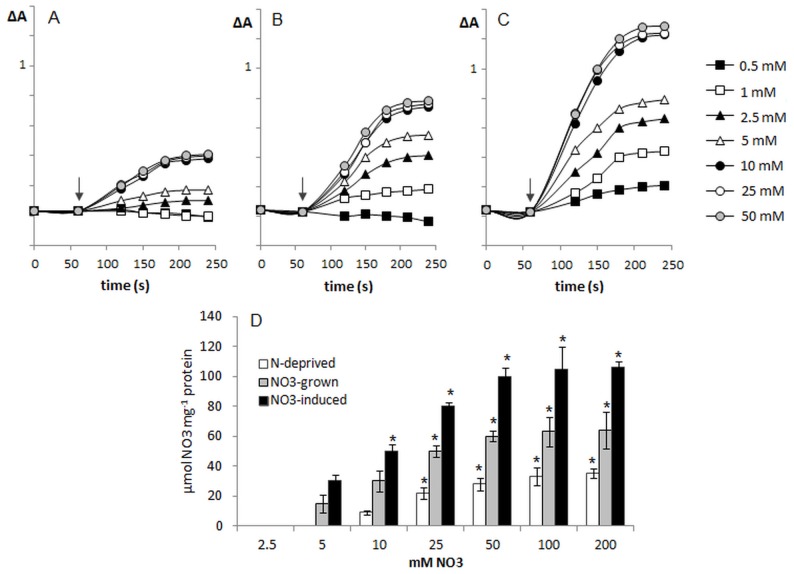
The activity of proton-coupled nitrate transport in the tonoplast membranes isolated from cucumbers grown under different nitrate supply. 50 µg of tonoplast membrane protein was incubated with 20 mM Tris-Mes 0,25 M sucrose, 1 mM DTT and acridine orange until a stable baseline was reached (3–5 min). The increase in acridine orange absorbance in membranes isolated from N-deprived (A), NO_3_^−^-grown (B) and NO_3_^−^-induced (C) plants was initiated by the addition of different concentrations of KNO_3_ into the reaction media (indicated by the arrows) and monitored during the following 3 min. Presented values are representative for the results obtained in three to four independent experiments with each experiment done in triplicate. D. The rate of nitrate accumulation in tonoplast vesicles isolated from N-deprived (white bars), NO_3_^−^-grown (light grey bars) and NO_3_^−^-induced (dark bars) plants determined by HPLC. The reaction mixture for HPLC assay was deprived of pH-sensitive probe. Values are the means ±SE (*n* = 5–6 measurements from 4–6 independent tonoplast preparations). Asterisks indicate a significant difference (*P*<0.05) between proton-coupled nitrate transport activities in tonoplast membranes isolated from different plants.

**Figure 3:**
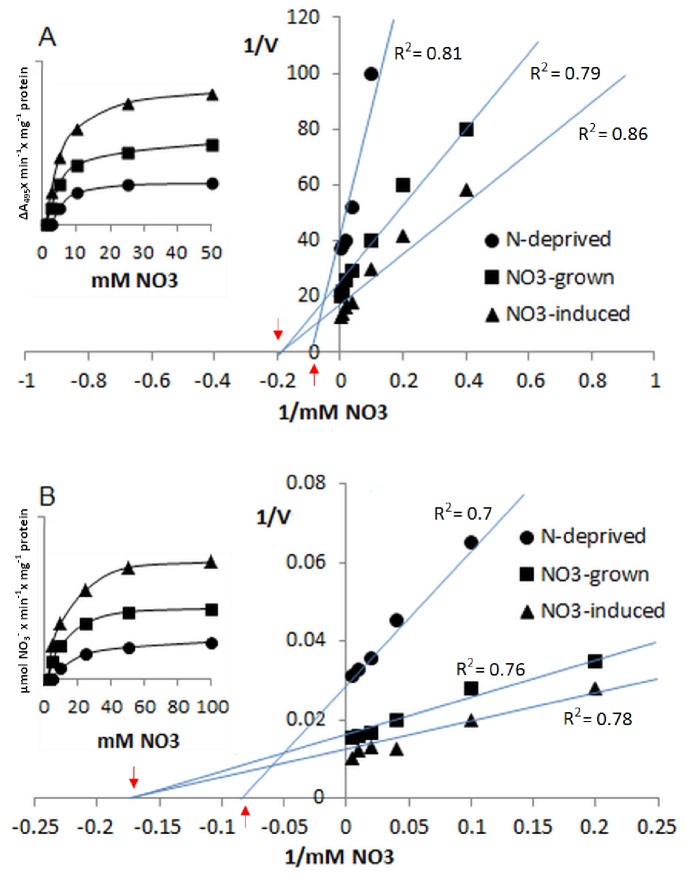
The kinetics of proton-coupled nitrate transport across the tonoplast membranes isolated from cucumbers grown under different nitrate supply. The effect of nitrate concentration on changes in acridine orange absorbance (A) or nitrate accumulation (B) in ΔpH-energized tonoplast vesicles isolated from N-deprived (circles), NO_3_^−^-grown (squares) and NO_3_^−^-induced (triangles) plants. The Km and R^2^ values were calculated using GraphPrism Software. The −1/Km values are indicated by red arrows. V represents the ΔA_495_×min^−1^×mg^−1^ protein (A) or the µmol NO_3_^−^×min^−1^×mg^−1^ protein (B).

The uptake of NO_3_^−^ by isolated tonoplast vesicles was measured either indirectly, through the measurement of proton fluxes reflecting nitrate antiport, or directly, by measurement of nitrate accumulation inside vesicles. In the assays employing pH-sensitive probes (acridine orange and quinacrine) different concentrations of KNO_3_ were added to the reaction media containing tonoplast membranes with imposed ΔpH and then acridine absorbance or quinacrine fluorescence was monitored for the next 3 minutes. The transport of nitrate into vesicles imposing a pH gradient caused an instant, ΔpH-dependent increase of quinacrine fluorescence (Figures S5A–C) or acridine absorbance (Figures 2A–C), which was not observed in the vesicles without transmembrane ΔpH (Figures S6A–F). The dissipation of ΔpH was dependent on both the source of tonoplast membrane and the concentration of nitrate added to the vesicles. As shown in Figures 2A–C and S5A–C, to initiate the increase of acridine orange absorbance or quinacrine fluorescence, different nitrate concentrations were required for the membranes obtained from plants differently supplied with nitrate. The first absorbance/fluorescence change induced by nitrate was observed at a similar 2.5 mM concentration for both membranes isolated from N-deprived and those from NO_3_^−^-grown roots, whereas in the case of tonoplast isolated from NO_3_-induced roots, lower nitrate concentration (0.5–1 mM) was sufficient to induce proton efflux from the vesicles. Tonoplast isolated from NO_3_^−^-induced roots displayed not only higher sensitivity but also higher capacity for nitrate transport, showing the maximum increase of absorbance/fluorescence approximately 2–3 times higher (100%), when compared with tonoplast isolated from N-deprived (35%) or NO_3_^−^-grown (60%) plants (Figures 2 and S5). Interestingly, the maximal rate of absorbance recovery for membranes isolated from plants growing under all nitrate regimes was obtained with 10 mM KNO_3_. Therefore, the active nitrate transport across tonoplast membranes from roots of NO_3_^−^-induced plants was induced at different but saturated at similar nitrate concentration compared to the relevant process observed in the membranes isolated from NO_3_^−^-uninduced plants. Since the phenomenon of nitrate transport into tonoplast vesicles exhibited different saturation kinetics in the membranes isolated from plants differently supplied with nitrate, the apparent Km values also varied in a way dependent on the membranes tested. However, Michaelis–Menten kinetics of nitrate transport were the same in the tonoplast of both NO_3_^−^-induced and NO_3_^−^-grown plants with the apparent Km of ∼5±1.2 mM (Figures 3A and S5D). In contrast, the nitrate antiport kinetics into membranes isolated from N-deprived plants were characterized by the apparent Km of ∼10±2.12 mM (Figures 3A and S5D). The results clearly show that the overall kinetics of acridine absorbance and quinacrine fluorescence recovery were different after the nitrate additions to the vesicles obtained from differently growing plants. In all the membranes the rate of NO_3_^−^-induced ΔpH dissipation was the highest during 120 s after addition of nitrate into the medium assay, and during the next 60 s the velocity of acridine absorbance increase was considerably lower (Figures 2A–C and S5A–C). Nevertheless, in the membranes obtained from N-deprived plant roots, the overall rate of nitrate transport was slower when compared to the vesicles obtained from NO_3_^−^-treated plants. The results of direct determination of nitrate accumulation within tonoplast vesicles measured by high-performance liquid chromatography clearly confirmed the output of assays employing pH-sensitive probes (Figure 2D). The highest and the lowest rate of nitrate accumulation occurred in membranes isolated from NO_3_^−^-induced and N-deprived plants, respectively. Nitrate accumulation within the membranes was also concentration-dependent and saturable, showing the features of Michaelis–Menten kinetics with Km ∼6±1.6 mM and ∼12±1.9 mM for tonoplast obtained from NO_3_^−^-treated and N-deprived plants, respectively (Figure 3B).

### Uptake of Cl^−^ and SO_4_^−^ into Tonoplast Vesicles

**Figure 4:**
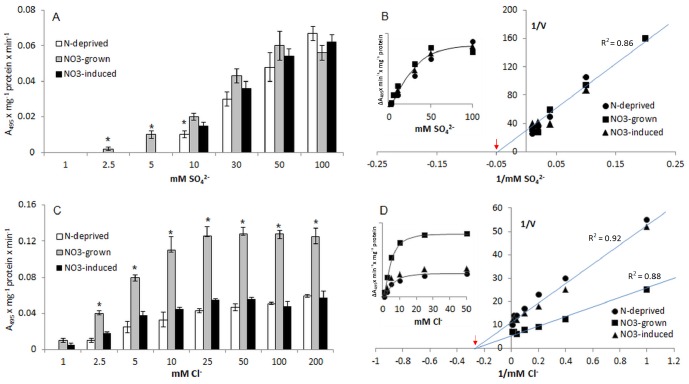
The properties of H^+^-coupled transport of sulphate and chloride in tonoplast vesicles isolated from the roots of cucumbers grown under different nitrate supply. The time-course of acridine orange absorbance change after the addition of different concentration of K_2_SO_4_ (A) or KCl (C) to the reaction media containing ΔpH-energized tonoplast vesicles obtained from NO_3_^−^-grown (grey bars), NO_3_^−^-induced (dark bars) or N-deprived (white bars) plants. The effect of sulphate (B) or chloride (D) concentration on the acridine orange absorbance ΔpH-energized, tonoplast obtained from NO_3_^−^-grown (squares), NO_3_^−^-induced (triangles), or N-deprived (circles) plants. Values are the means ±SE (*n* = 5–6 measurements from 4–6 independent tonoplast preparations). Asterisks indicate a significant difference (*P*<0.05) between H^+^-coupled SO_4_^2−^ and Cl^−^ transport in tonoplast isolated from different plants. The Km and R^2^ values were calculated using GraphPrism Software. The −1/Km values are indicated by red arrows. V represents the ΔA_495_×min^−1^×mg^−1^ protein.

To determine whether the observed nitrate antiport with its characteristics is selective only for nitrate ions we used potassium chlorate and potassium sulfate instead of potassium nitrate in the assay. As shown in Figure 4, the influx of Cl^−^ and SO_4_^−^ into tonoplast vesicles isolated from plants grown under different nitrate regimes could also occur through a ΔpH-dependent antiport system. However, the kinetic profiles of NO_3_^−^, Cl^−^ and SO_4_^−^ antiport were clearly different. Similarly to nitrate antiport, sulfate H^+^-coupled influx into membranes was concentration dependent, and saturable at 50–100 mM SO_4_^−^ concentration (Figures 4A and 4B). The overall kinetics of sulfate transport in all tonoplast membranes exhibited highly comparable features with the same apparent Vmax and Km of ∼20±2.8 mM (Figures 4A and 4B). In comparison, H^+^-coupled Cl^−^ transport into the vesicles was concentration dependent only in the lower range of external Cl^−^ concentration (1–5 mM), showing the features of Michaelis–Menten kinetics with the same apparent Km of ∼3±2.1 mM in all membranes (Figures 4C and 4D). Contrary to sulfate antiport activity, the Vmax of chloride transport was different under varying nitrate regimes, showing the highest value (the highest capacity for Cl^−^ influx) in tonoplast isolated from plants grown under constant NO_3_^−^ supply and the same lowest value in membranes obtained from N-deprived or NO_3_^−^-induced plants (Figure 4D). Hence, it may be assumed that the NO_3_^−^, Cl^−^ or SO_4_^−^ antiport in tonoplast membranes is mediated by different transport proteins or by a protein with different affinity and selectivity to these ions.

### The Effect of Cytosolic Fraction, Protein Kinase and Phosphatase Inhibitors and EGTA on the Proton and Nitrate Transport across Tonoplast Membranes

**Figure 5:**
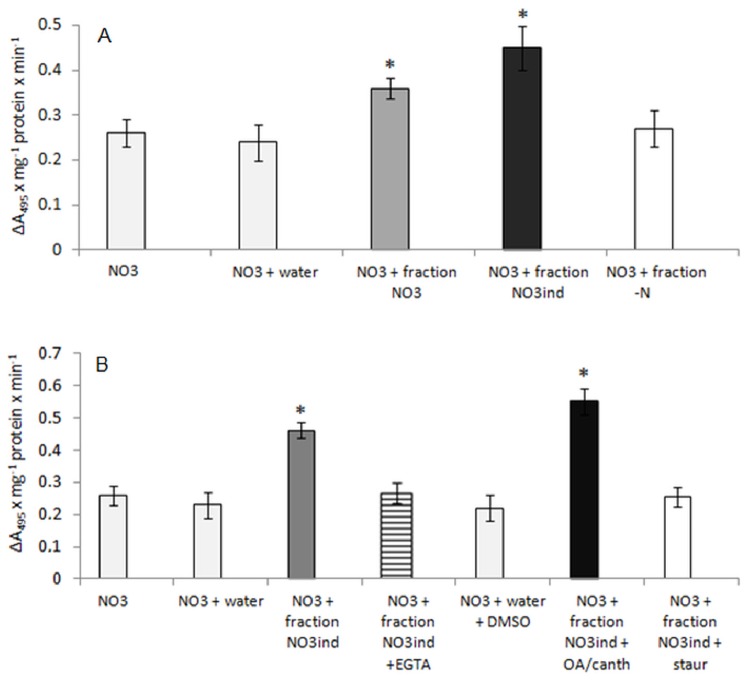
The effect of soluble fractions (A) and soluble fraction isolated from NO_3_ ^−^-**induced roots, protein kinase and phosphatase inhibitors or EGTA (B) on proton-coupled nitrate transport in tonoplast membranes obtained from NO_3_** ^−^-**grown plant roots.** Cytosolic soluble fraction (supernatant 120 000 g, 50 µl) alone or with other compounds was added to the reaction media containing 0.25 M sucrose, 1 mM DTT, 10 µM acridine orange and tonoplast membranes (50 µg of protein). After 5-min long incubation, 10 mM KNO_3_ was introduced into the membranes to initiate proton efflux from the vesicles observed as the acridine orange absorbance increase. At first, the rate of H^+^-coupled nitrate antiport was measured in the presence of KNO_3_ (light grey bars) and cytosolic fractions isolated from NO_3_^−^-grown (dark grey bars), NO_3_^−^-induced (dark bars) or N-deprived (white bars) plants (A). In further experiments, H^+^/NO_3_^−^ activity was also determined in the presence of KNO_3_, cytosolic fraction obtained from NO_3_^−^-induced plants and phosphatase inhibitors (black bars) or kinase inhibitor (white bars) or EGTA (striped bars) (B). Protein kinase inhibitor, staurosporine and phosphatase inhibitors, okadaic acid (OA) and cantharidin were used at 5 µM and 2 µM concentration, respectively, whereas EGTA was applied to the media at final 5 mM concentration. In control assays, equal amounts of water or DMSO was used instead of cytosolic fraction/EGTA or inhibitors, respectively (light grey bars). Values are the means ±SE (*n* = 5–6 measurements from 4–6 independent tonoplast preparations). Asterisks indicate a significant difference (*P*<0.05) between H^+^-coupled NO_3_^−^ transport in tonoplast isolated from different plants.

The increased nitrate antiport across root tonoplast membranes isolated from NO_3_^−^-induced plants could be attributed to NO_3_^−^-dependent post-translational modification of nitrate transport protein localized to the tonoplast membranes. Phosphorylation/dephosphorylation events catalyzed by protein kinases and protein phosphatases are the most common reverse modulations of protein activities in plant cells. Therefore, we have investigated whether protein kinases or phosphatases could be involved in the vacuolar nitrate antiport stimulation in NO_3_^−^-induced plants. For this purpose, the effect of soluble cytosolic fractions isolated from the roots of plants grown under different nitrate regimes on the NO_3_^−^/H^+^ transport activity in tonoplast obtained from the roots of NO_3_^−^-grown plants was studied. The soluble fraction was introduced into the reaction media at the beginning of 5-min incubation of membranes in the reaction mixture containing acridine orange. The addition of cytosolic fractions obtained from NO_3_^−^-grown or NO_3_^−^-induced plants to the assay resulted in over 30% and over 65% stimulation of nitrate antiport activity, respectively (Figure 5A). In contrast, the soluble fraction obtained from N-deprived plants did not affect H^+^/NO_3_^−^ transport significantly (Figure 5A). Due to its higher stimulatory effect, the cytosolic fraction obtained from NO_3_^−^-induced plants was used in the further studies on the posttranslational modulation of nitrate antiport activity. Specific inhibitors of protein kinases (staurosporine) and phosphatases (okadaic acid or cantharidin), as well as the calcium chelator EGTA, were added to the reaction media along with the cytosolic fraction in order to determine which component of the fraction affects nitrate antiport activity. Both phosphatase inhibitors, okadaic acid and cantharidin, enhanced the stimulatory effect of the cytosolic fraction on tonoplast H^+^/NO_3_ activity by over 30% (Figure 5B). In contrast, the unspecific protein kinase inhibitor staurosporine as well as calcium chelating EGTA completely abolished the positive effect of the soluble fraction on H^+^/NO_3_^−^ transport in the tonoplast (Figure 5B). The results suggest that Ca-dependent, staurosporine-sensitive protein kinase could be involved in the regulation of the vacuolar nitrate accumulation processes.

Since sufficient ΔpH across the tonoplast membrane is required for nitrate antiport activity, the effect of soluble cytosolic fractions on V-ATPase-mediated proton transport was also studied to establish whether the two closely linked activities (proton pumping into the vacuole and nitrate/proton exchange) undergo a similar regulation mode. Contrary to nitrate antiport activity in the tonoplast, none of the soluble fractions obtained from plants grown under different nitrate regimes affected proton pumping by V-ATPase (Figure S7).

### *CsCLCa*, *CsCLCc* and *CsCLCg* Expression in Cucumber Roots under Different Nitrate Regimes

**Figure 6:**
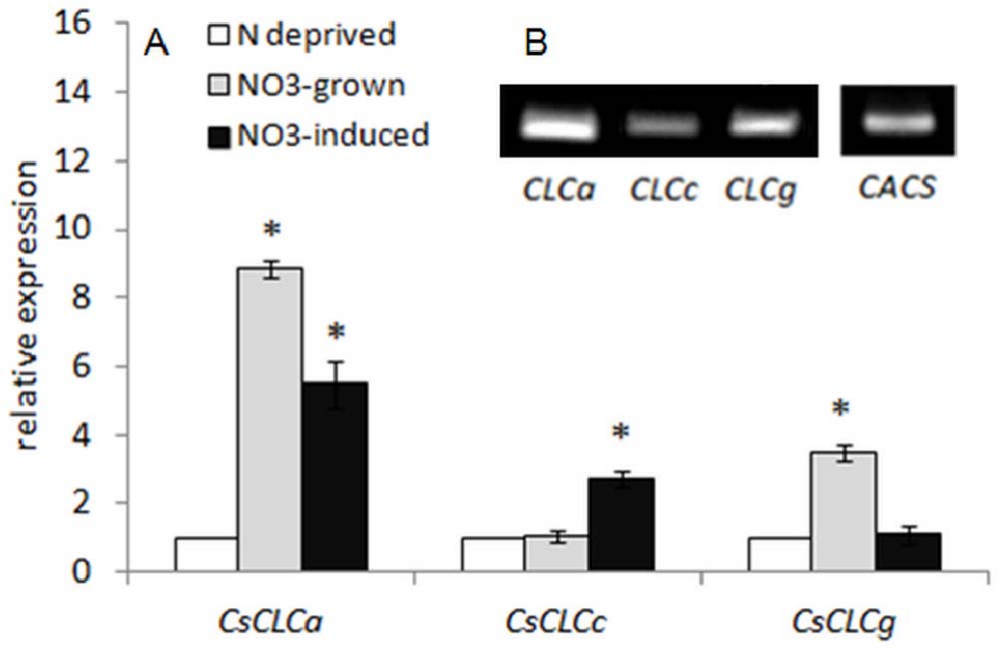
CsCLCs expression in roots of cucumber grown under different nitrate supply. The level of *CsCLCa*, *CsCLc* and *CsCLCg* transcript was analyzed by Real-time PCR (A) and PCR (B) using reversed transcribed total RNA isolated from plant roots. For the real-time PCR assay, total RNA was prepared from NO_3_^−^-grown (grey bars), NO_3_^−^-induced (dark bars) and N-deprived (white bars) plants. The cDNA synthesized from RNA isolated from NO_3_^−^-grown plants was also used for the PCR assay (B). The gene encoding clathrin adaptor complex subunit (CACS) was used as internal control. Values are the means ±SE (*n* = 5–6 measurements from 3 independent experiments). Asterisks indicate a significant difference (*P*<0.05) between the expression of *CsCLCs* under different availability of nitrate in nutrient solution (A). Presented picture (B) is representative for the results obtained in three to four independent experiments.

Four of the seven *Arabidopsis* CLCs have been localized to the tonoplast membrane: CLCa, CLCb, CLCc and CLCg. Only three homologs of tonoplast AtCLCs have been found in cucumber and were designated CLCa, CLCc and CLCg based on the homology to *Arabidopsis* proteins (Bogusz and Kłobus, unpublished; Lange and Kłobus, unpublished). The genes encoding three cucumber CLCs were differentially expressed in roots (Figures 6A and 6B). The *CLCa* expression was considerably stimulated under constant (over 8-fold) and temporary (over 5-fold) nitrate provision when compared to the level of transcript of the gene under nitrogen deficiency (Figure 6A). Similarly, *CLCg* mRNA was the most abundant in roots of cucumbers grown under constant nitrate supply, but contrary to *CLCa*, the expression of *CLCg* in NO_3_^−^-induced and N-deprived plants was comparable (Figure 6A). In contrast, *CLCc* transcript significantly increased only upon short-term nitrate provision (Figure 6A).

## Discussion

It is well evidenced that plants can develop considerably high levels of nitrate within vacuoles using proton-coupled nitrate transporters. Recent years have brought growing information regarding the molecular features and properties of the nitrate antiport system operating at the tonoplast. Active nitrate accumulation within vacuoles allows for storage of large amounts of nitrate inside the cell that can be remobilized during nitrate starvation. Vacuolar nitrate may also serve as an osmoticum at low light conditions to compensate for the shortage of carbohydrates resulting from decreased photosynthesis [48]. Although nitrate antiporter activity has been demonstrated at the tonoplast and protein candidates for nitrate antiporters have been proposed, the mechanisms of the regulation of active nitrate accumulation within vacuoles, with some exceptions, have not been revealed yet. Using tonoplast membranes isolated from cucumber roots grown in nitrate, we previously demonstrated that tonoplast nitrate antiport utilizes energy from the ATP-generated transmembrane proton gradient [28]. In this study we focus on elucidation of the regulation of the tonoplast H^+^/NO_3_^−^ antiport activity in response to external nitrogen (NO_3_^−^) nitrate availability.

### H^+^/NO_3_^−^ Antiport Activity Depends on the Availability of External Nitrate

The H^+^-coupled NO_3_^−^ antiport is closely connected to ΔpH generation across the tonoplast membrane catalyzed by V-ATPase. The assays using acridine orange as a ΔpH-sensitive dye showed that both V-ATPase-mediated and NO_3_^−^-induced proton movement across the tonoplast were significantly dependent on the availability of nitrate in the external solution (Figures 1 and 2). Nitrate is a commonly used V-ATPase inhibitor since it uncouples V-ATPase-mediated H^+^ pumping from ATP hydrolysis. Nevertheless, nitrogen deprivation of plants resulted in the significant decrease of H^+^-pumping and hydrolytic activity of the tonoplast proton pump (Figures 1A–C). This is not surprising since nitrogen is regarded as a major micronutrient required for the synthesis of crucial metabolites within plant cells, including amino acids, peptides, proteins, nucleic acids, etc. Hence, the biosynthesis of a large multimeric tonoplast proton pump should strictly depend on the nitrogen status of plant cells. Indeed, immunodetection of subunit a of tonoplast V-ATPase in membranes isolated from plants grown under different nitrogen regimes clearly confirmed that the lower V-ATPase activity in N-deprived plants could be a consequence of the decreased biosynthesis of proton pump subunits during N starvation (Figure 1E). However, though the level of V-ATPase protein was also decreased in NO_3_^−^-induced plants, the proton transport activity of this pump was significantly increased, suggesting the change in the coupling of proton transport and ATP hydrolysis of V-ATPase upon short-term nitrate provision. It has previously been reported that the efficiency of coupling may be increased (by Cu ions, transmembrane ΔpH decrease) or decreased (by Cd ions), affecting vacuolar acidification [31], [49], [50]. Hence, short-term (24 h) nitrate provision might have resulted in the change of cytoplasmic and vacuolar pH (decrease of ΔpH between cytosolic and vacuolar compartment), leading to the increased coupling efficiency of tonoplast V-ATPase and enhanced vacuolar acidification. Similar, nitrate-dependent changes in coupling efficiency of tonoplast V-ATPase were reported earlier in tobacco plants grown under varied nitrate nutrition [51]. High nitrate concentrations (10 mM, 20 mM) in the nutrition media caused higher rates of ATP hydrolysis and relative rates in proton transport activity when compared to the effect of 2 mM nitrate or ammonium as a nitrogen source [51]. As a result, relative coupling ratios were higher at the higher NO_3_^−^ concentrations [51].

The different rate of V-ATPase-dependent proton gradient generation could not provide the same level of proton motive force required for tonoplast antiport activities; hence an artificial ΔpH was imposed on the membranes to eliminate the effect of the transmembrane gradient on H^+^-coupled nitrate transport. Interestingly, nitrate antiport activity was detectable in plants cultivated in all nutritional conditions, though it was considerably lower in N-deprived plants (Figures 2 and S5). Hence, the decreased V-ATPase synthesis in N-deprived plants may result from the decreased activities of the proteins utilizing V-ATPase-catalyzed proton motive force formation. Such co-regulation of the proton pump and antiport activities at the tonoplast was previously demonstrated in mutants unable to synthesize CAX metal/H^+^ antiporters. The *CAX1* knock-out mutants not only exhibit a 50% reduction in vacuolar H^+^/Ca^2+^ antiport activity but also a 40% decrease in V-ATPase activity [52]. Thus both activities, ATP-dependent proton pumping and H^+^-coupling across tonoplast membranes, appear to be closely related and influence each other. Interestingly, similar correlation between proton pumping and nitrate transport activities was found at plasma membranes. As already demonstrated in maize and cucumber, the higher level of nitrate uptake by root [10], [11], [53] and leaf cells [53] was related with a significant increase in the biosynthesis and activity of PM H^+^-ATPase. Therefore, it has been suggested that nitrate may be the signal triggering the expression of the members of PM H+-ATPase subfamily resulting in the enhanced proton-pumping activity under increased nitrate inflow [11].

The properties of active nitrate transport into the tonoplast were strongly dependent on the nitrogen (NO_3_^−^) availability. Despite the lack of nitrogen in nutrient solution, the active nitrate antiport still operated at tonoplast membranes, suggesting that a constitutive, H^+^-coupled transport system with low capacity and affinity to NO_3_^−^ ions is present in the vacuolar membrane of plant cells (Figures 2 and S5). The H^+^/NO_3_^−^ antiport activity in plants treated with nitrate displayed significantly higher affinity and capacity for NO_3_^−^ ions, indicating that the existing nitrate antiport system was modified or an additional nitrate antiport system(s) with distinct kinetic properties operates in the vacuolar membranes of plants grown under nitrate availability. Interestingly, the overall nitrate antiport activity at the tonoplast was the highest in NO_3_^−^-inducible plants, suggesting that vacuoles were much more capable of active accumulation of nitrate ions after 24 hours of nitrate treatment than after 7 days of nitrate nutrition or deprivation. Such increased NO_3_^−^/H^+^ activity might have caused the decrease of ΔpH across the tonoplast membrane followed by the increase in the coupling efficiency of tonoplast V-ATPase (Figures 1A–C). The striking differences in nitrate antiport activity of tonoplast membranes isolated from roots of cucumbers grown under various nitrate availability may result from the morphological and developmental differences between differentially grown plants. Since only plants constantly supplied with nitrate developed the first leaf, the nitrate trafficking and its direction within the tissues of NO_3_^−^-induced and NO_3_^−^-grown plants were probably quite distinct. The leaf developed in NO_3_^−^-grown plants could serve as a sink for nitrate, since leaves play a significant role in nitrogen metabolism. It is known that of the whole absorbed nitrogen, only some is assimilated into amino acids in roots, whereas the majority seems to be translocated to the leaves in the transpiration stream. Consequently, most amino acid synthesis occurs in leaves [54]. This may explain why the tonoplast H^+^/NO_3_^−^ antiport activity in roots of NO_3_^−^-grown plants was considerably lower from the relevant activity observed in the NO_3_^−^-induced, leaf-deprived plants. The nitrogen (NO_3_^−^)-induced plants were morphologically similar to N-deprived plants, so after the transfer into nitrate-enriched medium following 6-day long N deprivation, they could accumulate higher nitrate levels in root cell vacuoles for storage and further remobilization during NO_3_^−^-induced increase of plant growth and development.

### The H^+^/NO_3_^−^ Antiport may be Subjected to Phosphorylation in Response to NO_3_^−^ (N) Induction

In plants, reversible protein phosphorylation seems to be the most common mechanism of post-translational modification of protein activity, ensuring rapid and reversible changes in the ability of plants to respond to specific stimuli. Nitrogen constitutes the major macronutrient limiting plant growth and development; thus plants had to develop mechanisms ensuring the rapid response and adaptation to frequent changes in the external nitrogen status. We have therefore investigated whether the increased nitrate antiport at tonoplast membranes isolated from N-induced plants may result from the posttranslational modification of antiporters occurring in response to 24-hour-long NO_3_^−^ induction. Soluble cytosolic fractions were used as the source of protein kinases or protein phosphatases that could be activated or synthesized under different NO_3_^−^ availability. The results show that the soluble fraction isolated from NO_3_^−^-treated plants affected H^+^/NO_3_^−^ antiport activity but not V-ATPase activity in membranes isolated from roots of NO_3_^−^-grown plants (Figures 5A and S7), indicating that the stimulation of both tonoplast activities, V-ATPase-mediated proton transport and H^+^-coupled NO_3_^−^ antiport, occurs via different regulation modes. Contrary to this, the plasma membrane proton pump was significantly stimulated by the cytosolic fraction isolated from the roots of NaCl-treated plants due to the increased phosphorylation of the membrane protein [55]. It should not be surprising, though, that these two proteins of strikingly different structure, composition and origin are regulated via distinct molecular mechanisms. Since phosphatase inhibitors (okadaic acid or cantharidin) enhanced and protein kinase inhibitors or EGTA completely abolished the stimulatory effect of the cytosolic fraction on active nitrate influx into membranes (Figure 5B), it may be suggested that stimulation of H^+^/NO_3_^−^ antiport activity in the roots of NO_3_^−^-induced plants results, at least partially, from the increased phosphorylation of the antiporters occurring during nitrogen (NO_3_^−^) supply. To our knowledge, this is the first evidence for the regulation of active accumulation of nitrate within the vacuole through the phosphorylation of nitrate transport proteins. It has been previously shown that the plasma membrane NRT1.1 transporter involved in nitrate uptake undergoes posttranslational regulation via phosphorylation or dephosphorylation events [56]. Low nitrate-induced phosphorylation of the threonine 101 within the NRT1.1 protein results in a switch from the low- to high-affinity mode of nitrate uptake by NRT1.1 [5]. The process is catalyzed by calcineurin B-like interacting protein kinase CIPK23 and enables the plant not only to transport nitrate into the cell but also to sense and detect the external nitrate level [6], [16], [18], [57]. Dephosphorylation of NRT1.1 protein in response to high nitrate concentration results in reversal of the protein affinity and thus ensures an efficient response of the plant to broad concentrations of external nitrate, allowing rapid adaptation to the environmental variability in soil nitrate levels. CIPKs are activated by calcium ions via the calcineurin B-like Ca-binding proteins and the resultant CBL–CIPK complexes appear to be critical for stress responses, potassium shortage, and for nitrate sensing [6], [58]–[64]. Evidence for nitrate-induced triggering of a calcium signal within the cell is still lacking but the results of our experiments indicate the requirement of calcium for nitrate-induced increase of the vacuolar H^+^/NO_3_^−^ transport related to phosphorylation of the antiporter. EGTA is a commonly known calcium chelator, so it could have abolished the stimulatory effect of the soluble fraction on tonoplast H^+^/NO_3_^−^ antiport activity through the binding of calcium, and thus decreasing the concentration of free calcium ions in the reaction media.

Summing up, the presented data indicate that the stimulation of H^+^/NO_3_^−^ antiport activity at tonoplast membranes of cucumber roots in response to short-term nitrate supply may occur through the phosphorylation of the membrane antiporter involving Ca-dependent, staurosporine-sensitive protein kinase. It remains to be elucidated, however, whether the protein kinase inducing the active nitrate accumulation within the vacuole belongs to the CIPK family of protein kinases activated by calcineurin B-like protein in a calcium-dependent manner. Since nitrate was the only source of nitrogen for plants in our experiments, it also remains to be elucidated whether the observed effect of different nitrate availability on active nitrate transport into the vacuole is nitrate specific, or results from alterations in the nitrogen status of plant cells.

### Proteins that could be Responsible for the H^+^/NO_3_^−^ Antiport at Tonoplast Membranes

In this work, we present evidence for the presence of distinct, NO_3_^−^-responsive antiport systems specific for nitrate in the tonoplast of cucumber root cells. For the last decades much attention has been given to identification of proteins that could mediate nitrate traffic and distribution within plant cells. In comparison to plasma membrane-localized NO_3_^−^ transporters, knowledge of the molecular identity and functions of vacuolar nitrate transporters is still very limited and mainly restricted to the chloride channel (CLC) family. Four of seven CLC proteins in *A. thaliana* were reported in tonoplast membranes: CLCa-c and CLC-g [65]. All Arabidopsis CLC genes are ubiquitously expressed throughout the plant with AtClCa showing the highest expression level [66]. Using electrophysiological and genetic approaches, it was demonstrated that AtCLC-a functions as a 2NO_3_^−^/1H^+^ exchanger that is able to accumulate nitrate in the vacuole [37]. AtClCa transcript levels were also shown to be up-regulated by nitrate in roots and shoots [67]. CLC-c might be another contributor of nitrate transport across the tonoplast, since the *atclc-c* mutant accumulated lower nitrate concentrations when grown at a range of external nitrate levels [68]. Contrary to *AtCLC-a*, the expression of AtCLC-c was down-regulated in the presence of nitrate [68]. CLC-b, the closest relative of CLC-a, was also shown to be a tonoplast H^+^-coupled NO_3_^−^ transporter; however, the *clc-b* knockout mutants displayed the same phenotype and accumulated as much nitrate and chloride as the wild type plants [38]. Hence, it seems that only CLCa is critically involved in the nitrate storage in the vacuole of plant cells. The evidence for posttranslational modification of plant CLC proteins is scarce. It was only demonstrated that ATP reversibly inhibits AtCLCa by interacting with the C-terminal domain [69]. We have recently identified all but one gene (*CLCb*) encoding tonoplast-localized AtCLCs homologs in the cucumber genome (Bogusz and Kłobus, unpublished). Only *CsCLCc* expression was increased upon short-term nitrate provision (Figure 6A), indicating that the enhanced nitrate accumulation in NO_3_^−^-induced plants may result from nitrate-induced antiporter phosphorylation and increased CsCLCc synthesis in response to nitrate supply. Still, the *CsCLCa* transcript was the most abundant under nitrogen (NO_3_^−^) provision even if its expression was significantly lower when compared to the mRNA level upon constant NO_3_^−^ supply (Figure 6A). Nevertheless, it remains to be established whether CLCa or CLCc undergoes phosphorylation in response to nitrate or nitrogen to clarify whether these proteins are responsible for the phosphorylation-mediated enhanced nitrate accumulation in root cell vacuoles in response to short-term nitrogen (NO_3_^−^) provision.

Phosphorylation-coupled nitrate transport has been well evidenced for the NRT1.1 transporter. Since the protein kinase C recognition motifs have also been found in the N- and C-terminal domains of the HvNRT2.1 transporter [20], the phosphorylation events may be common for both NRT1 and NRT2 transporters. Until now, among all characterized NRT proteins only few has been shown to be localized to vacuolar membrane: AtNRT2.7 and PTR2-like transporters from *A. thaliana* (PTR4 and PTR6), barley (HvPTR2) and *Hakea actites* (HaPTR4) [39], [70]–[74]. Nevertheless, only AtNRT2.7 is able to transport nitrate; this NRT2-like transporter is responsible for nitrate accumulation in the vacuoles of reproductive organs and seeds of *A. thaliana* [39]. All the functionally characterized NRT1-like transporters with confirmed ability to transport nitrate localize to the plasma membrane (as reviewed by [18]). The screening of the whole-genome shotgun reads (GenBank) revealed lack of the *AtNRT2.7* homolog in cucumber genome (Lange and Kłobus, unpublished); hence we assume that either CLC protein or the NRT1-like transporter (a low affinity transporter) or both systems are responsible for the H^+^/NO_3_^−^ antiport at the tonoplast in cucumber root cells. We have recently identified 13 homologs of *AtNRT1* genes in cucumber that are differentially regulated by nitrate provision [75]. However, the subcellular localization and regulation of the CsNRT1 as well as the CsCLC proteins remain to be established.

## Supporting Information

Figure S1 **Effect of MgATP (A), V-ATPase inhibitors: KNO3 or bafilomycin and gramicidin or NH_4_Cl (B) on the proton flux assay in tonoplast vesicles.** A. To induce V-ATPase-mediated proton influx into tonoplast vesicles, 3 mM MgATP (+MgATP) or 3 mM MgSO4 (-MgATP) were added to the reaction media. The proton gradient generation occurred only in the presence of ATP and was immediately inhibited by bafilomycin, a specific inhibitor of tonoplast proton pump V-ATPase. Protonophore gramicidin (5 µM) or NH_4_CL (5 mM***)*** caused an instant increase in acridine orange absorbance at 495 nm indicating that the observed changes in the aborbance of the probe result from the changes in proton gradient generation (by MgATP) and recovery (by gramicidin or NH_4_Cl). B. Bafilomycin (500 nmol) and KNO3 (50 mM) prevent V-ATPase-mediated proton translocation when added to the reaction along with MgATP. MgATP or MgSO_4_ were added to the reactions containing vacuolar membrane vesicles (50 µg), acridine orange (10 µM), 20 mM TRIS-MES (pH 7.2), 0.25 M sucrose, 50 mM KCl, and 1 mM DTT that had been preincubated for 5 min at room temperature. The quenching of acridine orange absorbance at 495 nm was monitored as described in Materials and methods.
(DOC)

Figure S2 **Mechanism for the use of ΔpH sensing acridine orange to measure MgATP-dependent proton fluxes in isolated tonoplast vesicles.** Acridine orange is a weak hydrophobic permeant probe containing an amine group as a weak base [76], [77]. In the unprotonated form, acridine orange has the capacity for free movement across the membrane while protonation of the basic amine group prevents free transmembrane movement of the probe [77]. During 5-min long incubation of tonoplast vesicles with acridine orange (pH 7.5), the unprotonated probe (green circles) freely moves across the membranes until the balance between the interior and exterior of the vesicles is achieved. The addition of MgATP into tonoplast vesicles initiates the V-ATP-mediated proton influx into tonoplast vesicles and acidification of vesicle lumen [77]. Under these conditions the protonation of the probe occurs (red circles) which renders acridine orange positively charged. As a result, the protonated form of the probe accumulates in the interior of the tonoplast membranes as it cannot freely move outside the lumen [77]. The pH-dependent accumulation of acridine orange within tonoplast membranes results in a change in the absorbtion spectrum and is detectable as the decrease of probe absorbance at 495 nm [76], [77]. Following the inhibition of V-ATPase (by bafilomycin) and the addition of NO_3_^−^ ions into the reaction media, NO_3_^−^-mediated proton efflux from the tonoplast membranes results in the decrease of the proton gradient and unprotonation of the acridine orange accumulated within vesicles. As a result, the corresponding efflux of the probe from the membranes into external solution occurs coupled to the increase of the probe absorbance at 495 nm [77]. OA, acridine orange
(DOC)

Figure S3 **Mechanism for the use of ΔpH sensing acridine orange to measure ΔpH-dependent proton fluxes in isolated tonoplast vesicles.** Tonoplast vesicles resuspended in pH 6.0 (to impose transmembrane ΔpH) or 8.0 (to maintain equal pH in the interior and exterior of the vesicles) were introduced into the reaction media of pH 8.0 and incubated with acridine orange for 5 min. Protonated probe (red circles) accumulated within the acidic interior of the tonoplast membranes. Following the addition of NO_3_^−^ ions to the reaction, the NO_3_^−^-induced proton efflux from the tonoplast membranes resulted in unprotonation of the acridine orange (green circles), change in the absorption spectra and corresponding efflux of the probe outside tonoplast lumen detectable as the increase in the absorbance of the probe at 495 nm (Figures 2A–C). In vesicles without transmembrane ΔpH, the addition of NO_3_^−^ ions did not induce proton efflux from the vesicle lumen due to the lack of proton motive force. The lack of vesicle lumen acidification rendered the acridine orange unprotonated and prevented accumulation of the probe inside vesicles. Hence, the absorbance of the probe at 495 nm did not change (Figure S6 D–F). Similar assays were performed with quinacrine using fluorescence change as ΔpH indicator (Figures S5 and S6 A-C). OA, acridine orange.
(DOC)

Figure S4 **The quality and integrity of RNA isolated from cucumber roots.** Total RNA was extracted from the roots of cucumber plants grown in constant (NO_3_^−^-grown) and temporary 24-hour-long (NO_3_^−^-induced) nitrate supply or from plants cultivated without nitrogen source (N-deprived). Following 5-min-long denaturation at 65°C and 2-min-long cooling on ice, RNA samples were subjected to electrophoresis using 2100 Bioanalyzer (Agilent) and RNA kits provided by the manufacturer. L - RNA ladder
(DOC)

Figure S5 **Characteristics of NO_3_** ^−^-**dependent increase in quinacrine fluorescence.** A pH gradient (inside-acid) was imposed in tonoplast vesicles isolated from roots of N-deprived (A), NO_3_^−^-grown (B) and NO_3_^−^-induced (C) cucumbers. The increase of quinacrine fluorescence was initiated by the addition of KNO_3_ into the reaction media (indicated by the black arrows) and monitored during the following 3 min. Presented values are representative for the results obtained in three to four independent experiments with each experiment done in triplicate. D. The effect of nitrate concentration on the changes in quinacrine fluorescence in ΔpH-energized tonoplast vesicles isolated from N-deprived (circles), NO_3_^−^-grown (squares) and NO_3_^−^-induced (triangles) plants. The Km and R^2^ values were calculated using GraphPrism Software. The −1/Km values are indicated by red arrows. V represents the ΔF×min^−1^×mg^−1^ protein.
(DOC)

Figure S6 **The effect of NO_3_^−^ on the changes in quinacrine fluorescence (A–C) and acridine orange absorbance (D–F) in tonoplast membranes without transmembrane ΔpH.** Tonoplast vesicles were isolated from roots of N-deprived (A, D), NO_3_^−^-grown (B, E) and NO_3_^−^-induced (C, F) cucumbers. Following 5-min-long incubation of vesicles with pH-sensitive probes, the different concentrations of KNO_3_ were added into the reaction media (indicated by the arrows) and the acridine orange absorbance or quinacrine fluorescence were monitored during the following 3 min. Presented values are representative for the results obtained in three to four independent experiments with each experiment done in triplicate.
(DOC)

Figure S7 **The effect of soluble fractions obtained from NO_3_** ^−^-**induced roots, protein kinase and phosphatase inhibitors or EGTA on proton transport in tonoplast membranes isolated from NO_3_-grown plant roots.** Cytosolic soluble fraction (supernatant 120 000 g, 50 µl) alone or with other compounds was added to the reaction media containing 0.25 M sucrose, 1 mM DTT, 10 µM acridine orange and tonoplast membranes (50 µg of protein). After 5-min long incubation, MgATP was introduced into the membranes to initiate proton gradient formation and the acridine orange absorbance changes were monitored during the next three min at 495 nm. The rate of transmembrane ΔpH was measured in the presence of MgATP (light grey bars) and cytosolic fraction (dark grey bars) or cytosolic fractions and phosphatase inhibitors (black bars) or cytosolic fraction and kinase inhibitor (white bars). Protein kinase inhibitor, staurosporine and phosphatase inhibitors, okadaic acid (OA) and cantharidine were used at 5 µM and 2 µM concentration, respectively. In control assays, equal amounts of water or DMSO (light grey bars) was used instead of cytosolic fraction or inhibitors, respectively. Values are the means ±SE (*n* = 5–6 measurements from 4–6 independent tonoplast preparations).
(DOC)
