# Supplementary figures and images for: NO3 −/H+ Antiport in the Tonoplast of Cucumber Root Cells Is Stimulated by Nitrate Supply: Evidence for a Reversible Nitrate-Induced Phosphorylation of Vacuolar NO3 −/H+ Antiport
Source: PLoS One. 2013 Sep 11;8(9):e73972. doi: 10.1371/journal.pone.0073972 (PMC3770621; doi:10.1371/journal.pone.0073972)

SUPPORTING INFORMATION

Figure S1

**
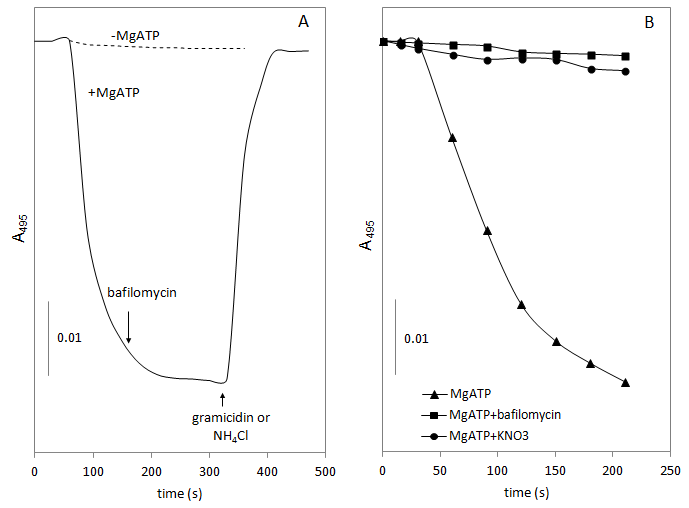
**

Supplement: Figure S1 — Effect of MgATP (A), V-ATPase inhibitors: KNO3 or bafilomycin and gramicidin or NH4Cl (B) on the proton flux assay in tonoplast vesicles. A. To induce V-ATPase-mediated proton influx into tonoplast vesicles, 3 mM MgATP (+MgATP) or 3 mM MgSO4 (-MgATP) were added to the reaction media. The proton gradient generation occurred only in the presence of ATP and was immediately inhibited by bafilomycin, a specific inhibitor of tonoplast proton pump V-ATPase. Protonophore gramicidin (5 µM) or NH4CL (5 mM) caused an instant increase in acridine orange absorbance at 495 nm indicating that the observed changes in the aborbance of the probe result from the changes in proton gradient generation (by MgATP) and recovery (by gramicidin or NH4Cl). B. Bafilomycin (500 nmol) and KNO3 (50 mM) prevent V-ATPase-mediated proton translocation when added to the reaction along with MgATP. MgATP or MgSO4 were added to the reactions containing vacuolar membrane vesicles (50 µg), acridine orange (10 µM), 20 mM TRIS-MES (pH 7.2), 0.25 M sucrose, 50 mM KCl, and 1 mM DTT that had been preincubated for 5 min at room temperature. The quenching of acridine orange absorbance at 495 nm was monitored as described in Materials and methods. (DOC) [file pone.0073972.s001.doc]

SUPPORTING INFORMATION

Figure S2


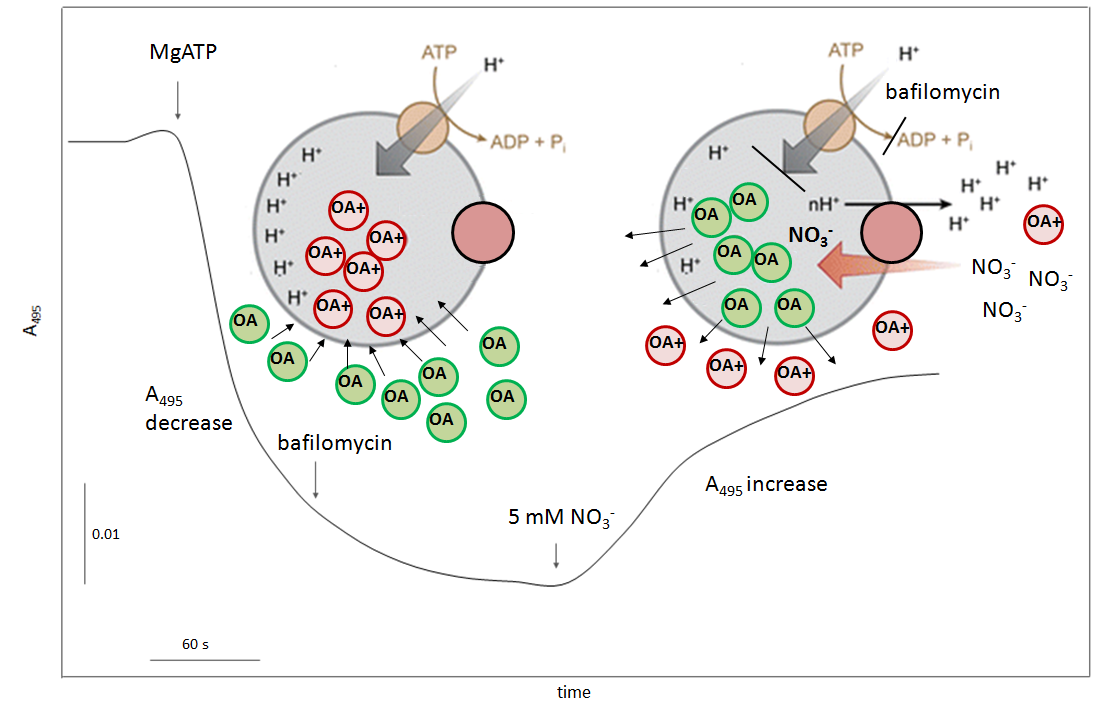

Supplement: Figure S2 — Mechanism for the use of ΔpH sensing acridine orange to measure MgATP-dependent proton fluxes in isolated tonoplast vesicles. Acridine orange is a weak hydrophobic permeant probe containing an amine group as a weak base [76], [77]. In the unprotonated form, acridine orange has the capacity for free movement across the membrane while protonation of the basic amine group prevents free transmembrane movement of the probe [77]. During 5-min long incubation of tonoplast vesicles with acridine orange (pH 7.5), the unprotonated probe (green circles) freely moves across the membranes until the balance between the interior and exterior of the vesicles is achieved. The addition of MgATP into tonoplast vesicles initiates the V-ATP-mediated proton influx into tonoplast vesicles and acidification of vesicle lumen [77]. Under these conditions the protonation of the probe occurs (red circles) which renders acridine orange positively charged. As a result, the protonated form of the probe accumulates in the interior of the tonoplast membranes as it cannot freely move outside the lumen [77]. The pH-dependent accumulation of acridine orange within tonoplast membranes results in a change in the absorbtion spectrum and is detectable as the decrease of probe absorbance at 495 nm [76], [77]. Following the inhibition of V-ATPase (by bafilomycin) and the addition of NO3 − ions into the reaction media, NO3 −-mediated proton efflux from the tonoplast membranes results in the decrease of the proton gradient and unprotonation of the acridine orange accumulated within vesicles. As a result, the corresponding efflux of the probe from the membranes into external solution occurs coupled to the increase of the probe absorbance at 495 nm [77]. OA, acridine orange (DOC) [file pone.0073972.s002.doc]

SUPPORTING INFORMATION

Figure S3

**
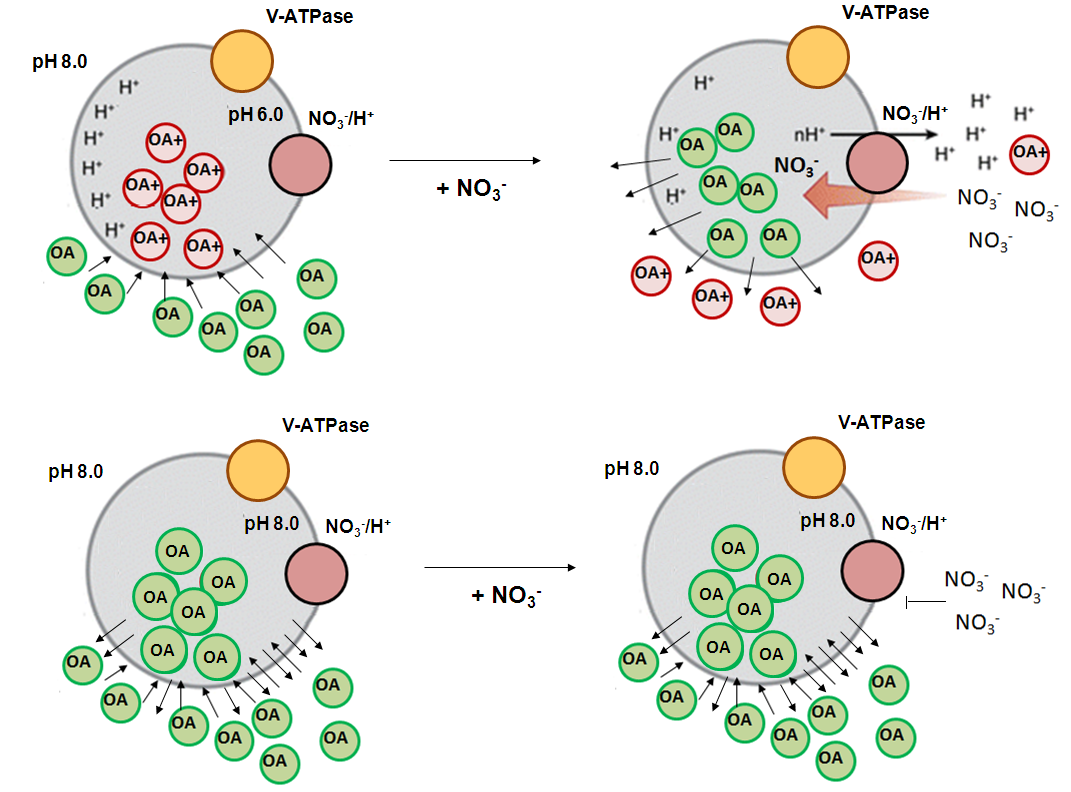
**

Supplement: Figure S3 — Mechanism for the use of ΔpH sensing acridine orange to measure ΔpH-dependent proton fluxes in isolated tonoplast vesicles. Tonoplast vesicles resuspended in pH 6.0 (to impose transmembrane ΔpH) or 8.0 (to maintain equal pH in the interior and exterior of the vesicles) were introduced into the reaction media of pH 8.0 and incubated with acridine orange for 5 min. Protonated probe (red circles) accumulated within the acidic interior of the tonoplast membranes. Following the addition of NO3 − ions to the reaction, the NO3 −-induced proton efflux from the tonoplast membranes resulted in unprotonation of the acridine orange (green circles), change in the absorption spectra and corresponding efflux of the probe outside tonoplast lumen detectable as the increase in the absorbance of the probe at 495 nm (Figures 2A–C). In vesicles without transmembrane ΔpH, the addition of NO3 − ions did not induce proton efflux from the vesicle lumen due to the lack of proton motive force. The lack of vesicle lumen acidification rendered the acridine orange unprotonated and prevented accumulation of the probe inside vesicles. Hence, the absorbance of the probe at 495 nm did not change (Figure S6 D–F). Similar assays were performed with quinacrine using fluorescence change as ΔpH indicator (Figures S5 and S6 A-C). OA, acridine orange. (DOC) [file pone.0073972.s003.doc]

SUPPORTING INFORMATION

Figure S4


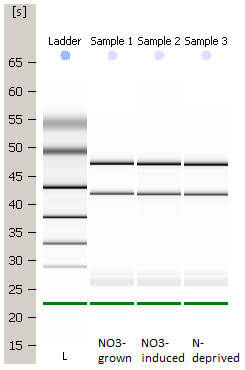

Supplement: Figure S4 — The quality and integrity of RNA isolated from cucumber roots. Total RNA was extracted from the roots of cucumber plants grown in constant (NO3 −-grown) and temporary 24-hour-long (NO3 −-induced) nitrate supply or from plants cultivated without nitrogen source (N-deprived). Following 5-min-long denaturation at 65°C and 2-min-long cooling on ice, RNA samples were subjected to electrophoresis using 2100 Bioanalyzer (Agilent) and RNA kits provided by the manufacturer. L - RNA ladder (DOC) [file pone.0073972.s004.doc]

SUPPORTING INFORMATION

Figure S5

**
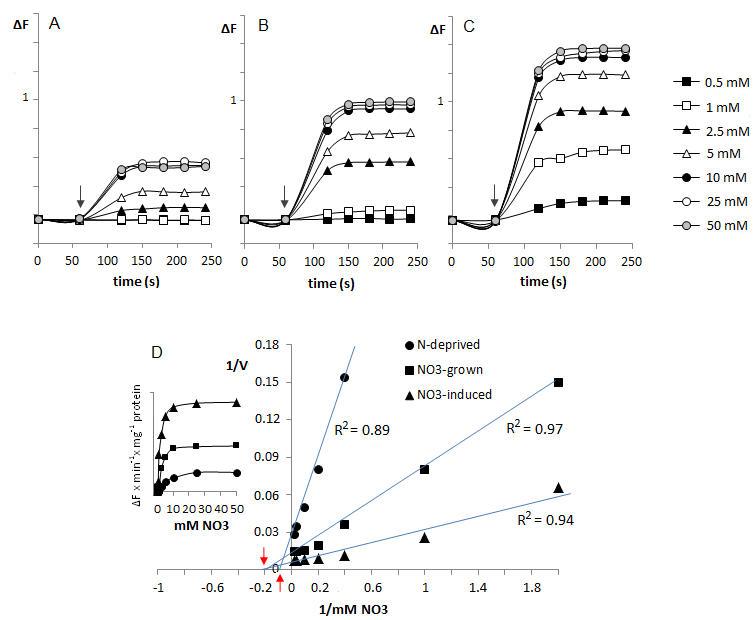
**

Supplement: Figure S5 — Characteristics of NO3 −-dependent increase in quinacrine fluorescence. A pH gradient (inside-acid) was imposed in tonoplast vesicles isolated from roots of N-deprived (A), NO3 −-grown (B) and NO3 −-induced (C) cucumbers. The increase of quinacrine fluorescence was initiated by the addition of KNO3 into the reaction media (indicated by the black arrows) and monitored during the following 3 min. Presented values are representative for the results obtained in three to four independent experiments with each experiment done in triplicate. D. The effect of nitrate concentration on the changes in quinacrine fluorescence in ΔpH-energized tonoplast vesicles isolated from N-deprived (circles), NO3 −-grown (squares) and NO3 −-induced (triangles) plants. The Km and R2 values were calculated using GraphPrism Software. The −1/Km values are indicated by red arrows. V represents the ΔF×min−1×mg−1 protein. (DOC) [file pone.0073972.s005.doc]

SUPPORTING INFORMATION

Figure S6

**
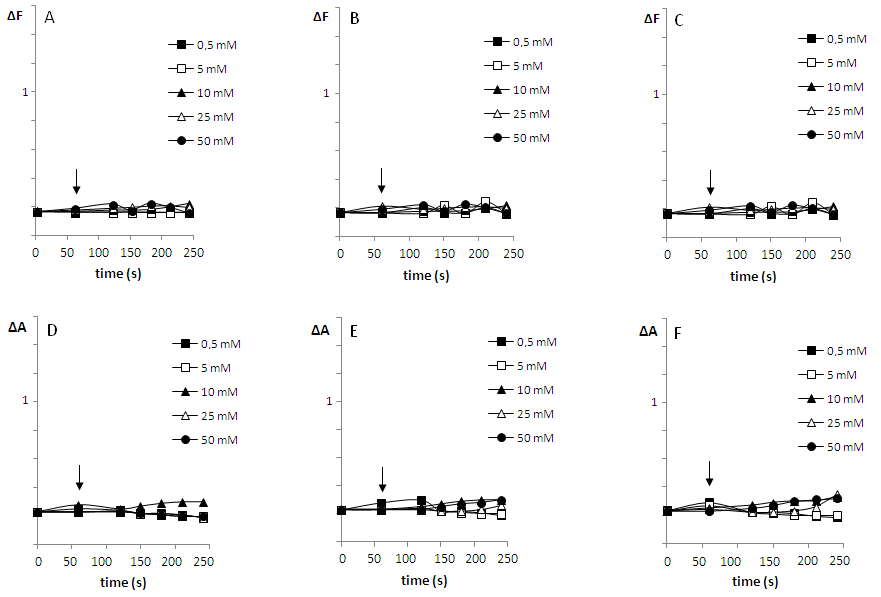
**

Supplement: Figure S6 — The effect of NO3− on the changes in quinacrine fluorescence (A–C) and acridine orange absorbance (D–F) in tonoplast membranes without transmembrane ΔpH. Tonoplast vesicles were isolated from roots of N-deprived (A, D), NO3 −-grown (B, E) and NO3 −-induced (C, F) cucumbers. Following 5-min-long incubation of vesicles with pH-sensitive probes, the different concentrations of KNO3 were added into the reaction media (indicated by the arrows) and the acridine orange absorbance or quinacrine fluorescence were monitored during the following 3 min. Presented values are representative for the results obtained in three to four independent experiments with each experiment done in triplicate. (DOC) [file pone.0073972.s006.doc]

SUPPORTING INFORMATION

Figure S7


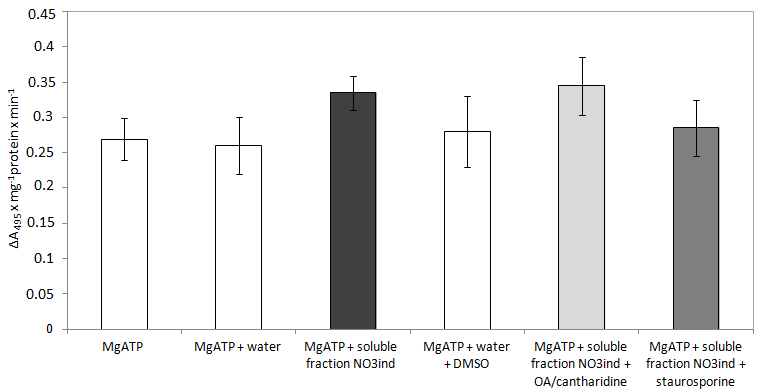

Supplement: Figure S7 — The effect of soluble fractions obtained from NO3 −-induced roots, protein kinase and phosphatase inhibitors or EGTA on proton transport in tonoplast membranes isolated from NO3-grown plant roots. Cytosolic soluble fraction (supernatant 120 000 g, 50 µl) alone or with other compounds was added to the reaction media containing 0.25 M sucrose, 1 mM DTT, 10 µM acridine orange and tonoplast membranes (50 µg of protein). After 5-min long incubation, MgATP was introduced into the membranes to initiate proton gradient formation and the acridine orange absorbance changes were monitored during the next three min at 495 nm. The rate of transmembrane ΔpH was measured in the presence of MgATP (light grey bars) and cytosolic fraction (dark grey bars) or cytosolic fractions and phosphatase inhibitors (black bars) or cytosolic fraction and kinase inhibitor (white bars). Protein kinase inhibitor, staurosporine and phosphatase inhibitors, okadaic acid (OA) and cantharidine were used at 5 µM and 2 µM concentration, respectively. In control assays, equal amounts of water or DMSO (light grey bars) was used instead of cytosolic fraction or inhibitors, respectively. Values are the means ±SE (n = 5–6 measurements from 4–6 independent tonoplast preparations). (DOC) [file pone.0073972.s007.doc]
